# Drug-Induced Stuttering: Occurrence and Possible Pathways

**DOI:** 10.3389/fpsyt.2021.692568

**Published:** 2021-08-25

**Authors:** Corine Ekhart, Florence van Hunsel, Peter van Harten, Jeanette van Baarsen, Tan Yingying, Bert Bast

**Affiliations:** ^1^Netherlands Pharmacovigilance Centre Lareb, 's-Hertogenbosch, Netherlands; ^2^Research Department, Psychiatric Centre GGz Centraal, Innova, Amersfoort, Netherlands; ^3^Department of Mental Health and Neuroscience, Faculty of Health Medicine and Life Sciences, Maastricht University, Maastricht, Netherlands; ^4^Centrum Voor Stottertherapie, Haarlem, Netherlands; ^5^Linguistic Institute, Shanghai International Studies University, Shanghai, China; ^6^StotterFonds, Nijkerk, Netherlands

**Keywords:** stuttering, adverse drug reaction, antiepileptics, antipsychotics, antidepressants, dopamine, serotonin

## Abstract

**Background:** Stuttering is a well-known condition that affects mainly children. Often, they recover as they get older. However, a drug-induced form of stuttering may occur at any age. The aim of the present study was to detect drugs that have been associated with stuttering and discuss the mechanisms involved.

**Method:** A descriptive study based on reports submitted to the global pharmacovigilance database VigiBase of the WHO was conducted.

**Results:** A total of 3,385 reports of dysphemia were retrieved from VigiBase. These reports were contributed by 51 countries. Antiepileptics, antidepressants, immunosuppressants, antipsychotics, and centrally acting sympathomimetics were among the most frequently implicated drugs.

**Conclusion:** A wide variety of drugs has been linked to the occurrence or recurrence of stuttering. Several mechanisms, such as increased dopamine levels, reduction of GABA, anticholinergic properties of drugs, or changes in serotonin levels, have been associated with the development of drug-induced stuttering. Paradoxically, agents known to reduce stuttering in some people may induce it in others.

## Introduction

Worldwide, about 80 million people (~1%, based on US data, which seem to apply worldwide) stutter, and therefore, it is a relatively common disorder. Stuttering is defined as a *disturbance in the normal fluency and time patterning of speech characterized by repetition of sounds, syllables, or words; prolongation of sounds; and interruptions in speech known as block*s (1). Developmental stuttering is the most common form and usually has a gradual onset. It occurs most often in children between the ages of 2 and 6 years, when children are developing their language skills. Contrary to this developmental stuttering, another form, i.e., acquired stuttering, can begin at any age and has a sudden onset. A sub-form of this, e.g., neurogenic stuttering, is almost always associated with brain function impairment due to a stroke, head trauma, or other type of brain injury (1).

Stuttering has also been described as an adverse drug reaction (ADR) associated with a variety of drugs. Whether it pertains to recurrence of developmental stuttering in the past or a *de novo* form is not always known. In the literature, drug-induced stuttering has been associated with several drugs such as antiepileptics, antidepressants, antipsychotics, and methylphenidate (2–4). Evidence that several drugs may induce stuttering in humans is based on case series limited in size.

The aim of the present study was to detect drugs that have been associated with stuttering (be it newly occurring or a recurrence of recovered developmental stuttering) by conducting a descriptive study based on reports submitted to the global pharmacovigilance database VigiBase of the WHO. Also, possible pathways are being discussed.

## Methods

Together with the Uppsala Monitoring Centre (UMC) in Sweden, the WHO promotes pharmacovigilance activities in countries all over the world. The UMC maintains the global adverse drug reaction (ADR) database of individual case safety reports (ICSRs) VigiBase. This is the largest pharmacovigilance database in the world, with over 23 million reports of suspected adverse effects of medicines, submitted since 1968 by over 130 countries (5). Data on ICSRs from VigiBase were extracted on April 3, 2020, by a data scientist of the UMC. The dataset date was March 29, 2020. The reported ADRs in VigiBase are coded with the Medical Dictionary for Regulatory Activities (MedDRA®). MedDRA® is a standardized international medical terminology that can be used for regulatory communication and evaluation of data pertaining to medicinal products for human use. There are five levels to the MedDRA hierarchy, arranged from a most specific term [the Lowest Level Term (LLT)] to very general terms [System Organ Class (SOC) term]. In this study, we used the level of “Preferred Terms” (PTs), that is, the second most specific term that is commonly used in pharmacovigilance to describe ADRs. A PT is a distinct descriptor (single medical concept) for a symptom, sign, disease diagnosis, therapeutic indication, investigation, surgical or medical procedure, and medical social, or family history characteristic (6). Drugs are coded according to the Anatomical Therapeutic Chemical (ATC) classification system. In the ATC classification system, the active substances are classified in a hierarchy with five different levels. In this study, data of the fourth level (therapeutical subgroup) and fifth level (chemical substance) were used.

The extracted dataset contained all ICSRs with a MedDRA PT dysphemia and drugs that were coded as suspected or interacting. The PT dysphemia encompasses several LLTs, e.g., stuttering, stammering, syllable stumbling. Demographic characteristics, number of reports, and disproportionality [Reporting Odds Ratio (ROR)] of associations were obtained using Vigilyze®, which is a search and analysis tool (available to member countries of the WHO Program for International Drug Monitoring). The ROR represents the extent to which the association between the ADR and suspect drug stands out with respect to its background frequency in the database. It is the odds of a certain event occurring with your medicinal product compared to the odds of the same event occurring with all other medicinal products in the database. If the ROR is statistically significant (lower limit 95% confidence interval >1), then the ADR is significantly associated with the suspect drug in reference to other reports in the database. It is expressed as a point estimate with corresponding 95% confidence intervals. At least three reports have to be present in the database to compute a reliable ROR. The ROR is a measure of disproportionality, not of causality. It is important to note that the information in VigiBase originates from multiple sources (different types of reporters and different countries), and the amount of information given as well as the likelihood that a drug caused the ADR may vary from case to case.

## Results

In total, 3,385 reports of dysphemia were retrieved from VigiBase, dating from June 30, 1988, to March 29, 2020. These reports were contributed by 51 countries. Most reports were submitted by consumers or other non-health professionals (*n* = 1,552, 46%), and health care professionals contributed 35%. For 655 reports (19%), the reporter was not known or more than one profession was mentioned. In the reports, 57% of the patients were female, 39% male, and in 4%, gender was unknown. The mean reported age was 39 years (median age 42 years). In 30% of the reports, age was unknown.

We found a significant disproportionality between drug use and stuttering for 104 drugs at the fifth ATC level, the chemical substance. The top 20 drugs associated with dysphemia, ranked according to highest disproportionality in VigiBase, are shown in Table 1. A high disproportionality (reported in Table 1) is not necessarily related to a high number of reports of dysphemia in the database. Table 2 shows the top 20 drugs associated with dysphemia, ranked according to the highest number of reports. In Table 3, the top 10 ranking for highest number of reports is shown on the level of the chemical subgroup (the fourth level).

**Table 1:** Top 20 drug-stuttering associations at the fifth ATC level ranked according to the highest disproportionality (ROR value) in VigiBase.

**Drug**	**Number of reports**	ROR^*^	**95% CI**
Tiagabine	4	25.6	9.6–68.3
Lomustine	4	24.0	9.0–64.1
Dexmethylphenidate	5	20.3	8.4–48.8
Ziconotide	5	15.7	6.5–37.8
Dosulepin	5	14.3	6.0–34.5
Lurasidone	19	14.1	9.0–22.2
Guanfacine	6	12.5	5.6–27.9
Topiramate	54	12.5	9.5–16.3
Lisdexamfetamine	24	12.3	8.2–18.3
Dobutamine	4	10.1	3.8–27.1
Brivaracetam	3	9.9	3.2–30.8
Dexamfetamine	4	9.7	3.7–26.0
Asenapine	10	9.6	5.2–17.9
Pregabalin	175	9.5	8.1–11.0
Atomoxetine	31	8.6	6.0–12.2
Methylphenidate	54	8.1	6.2–10.6
Cyclobenzaprine	9	7.8	4.1–15.0
Gabapentin	86	7.6	6.2–9.5
Montelukast	28	7.6	5.3–11.1
Bupropion	71	7.4	5.8–9.3

* *ROR, reporting odds ratio; the ROR is a measure of disproportionality. If the ROR is statistically significant (lower limit 95% confidence interval >1), then stuttering is significantly associated with the drug. 95% CI, 95% confidence interval; ATC, Anatomical Therapeutic Chemical*.

**Table 2:** Top 20 drugs at the fifth ATC level ranked according to the highest number of reports in VigiBase; the ROR is given as well.

**Drug**	**Number of reports**	ROR^*^	**95% CI**
Pregabalin	175	9.5	8.1–11.0
Natalizumab	162	7.1	6.0–8.3
Interferon beta-1a	119	4.5	3.8–5.4
Gabapentin	86	7.6	6.2–9.5
Quetiapine	75	6.3	5.0–7.9
Clozapine	75	3.3	2.6–4.1
Bupropion	71	7.4	5.8–9.3
Adalimumab	70	0.9	0.7–1.1
Duloxetine	67	6.9	5.4–8.7
Fumaric acid	60	3.6	2.8–4.6
Influenza vaccine	55	1.5	1.1–1.9
Topiramate	54	12.5	9.5–16.3
Methylphenidate	54	8.1	6.2–10.6
Venlafaxine	53	5.6	4.2–7.3
Lamotrigine	52	6.0	4.5–7.8
Olanzapine	50	5.1	3.9–6.8
Sertraline	50	4.6	3.4–6.0
Fingolimod	49	4.0	3.0–5.3
Aripiprazole	47	5.1	3.8–6.8
Fluoxetine	43	3.7	2.7–4.9

* *ROR, reporting odds ratio; the ROR is a measure of disproportionality. If the ROR is statistically significant (lower limit 95% confidence interval >1), then stuttering is significantly associated with the drug. 95% CI, 95% confidence interval; ATC, Anatomical Therapeutic Chemical*.

**Table 3:** Top 10 drug groups at the fourth ATC level ranked according to the highest number of reports in VigiBase.

**Drug**	**Number of reports**
Other antiepileptics	412
Selective immunosuppressants	253
Other antidepressants	224
Diazepines, oxazepines, thiazepines, and oxepines	210
Selective serotonin reuptake inhibitors	166
Interferons	157
Centrally acting sympathomimetics	132
Tumor necrosis factor alpha inhibitors	121
Other antipsychotics	107
Other immunosuppressants	77

*ATC, anatomical therapeutic chemical*.

In the top 20 drug-stuttering associations with the highest disproportionality (Table 1), five psychostimulants used for attention deficit hyperactivity disorder (ADHD) are present: dexmethylphenidate, lisdexamfetamine, dexamfetamine, atomoxetine, and methylphenidate. Furthermore, antiepileptics are frequently occurring in this top 20: tiagabine, topiramate, brivaracetam, pregabalin, and gabapentin.

Looking in another way, in the top 20 drug-stuttering associations with the highest number of reports (Table 2), it can be seen that besides the antiepileptics, antipsychotics (quetiapine, clozapine, olanzapine, and aripiprazole—all with a significant ROR), and antidepressants (bupropion, duloxetine, venlafaxine, sertraline, and fluoxetine—all with a significant ROR as well), also immunosuppressants/immunomodulating drugs (natalizumab, adalimumab, fingolimod, and interferon beta-1a) have a high number of reports of stuttering. The unexpected high ROR of natalizumab, fingolimod, as well as interferon beta-1a may relate to the (here not further explored) application in multiple sclerosis, a cerebral inflammation.

## Discussion

Controlled studies of drug-induced stuttering are not available, and only case reports (sometimes of larger size) have been reported. We analyzed the reported cases of drug-induced stuttering as extracted from the global ICSR database VigiBase.

When analyzing this dataset, searching for the PT dysphemia, one is struck with the variety of drugs reported to induce stuttering. Antiepileptics, antidepressants, immunosuppressants, antipsychotics, and centrally acting sympathomimetics are among the most frequently implicated drugs. The data are given ranked by ROR (Table 1) as well as ranked by number (Table 2).

### Case Reports

In the literature, drug-induced stuttering is reported as an ADR as a result of a change in the use of drugs of a patient. Stuttering as an ADR of medication is expected to show characteristics of the acquired type (7). Several drug classes are associated with the development of stuttering, mostly antidepressants, antiepileptics, and antipsychotics.

In a study of acquired stuttering in veterans of the wars, it was seen that over 66% of those with stuttering were prescribed at least one medication that may have affected speech fluency (antidepressants, anxiolytics, and antiepileptic drugs) compared with 35% of those without acquired stuttering (8).

A study by Bar et al. (9) describes a case series of seven patients aged 36–57 years who were treated with olanzapine or clozapine (both broad-spectrum antipsychotics) who began to stutter a few days to a few weeks after initiation. In six of the seven patients, this recovered rapidly after drug withdrawal (within 2–4 days). One patient had a medical history of stuttering. The other patients had no medical history of speech problems. Paradoxically, for olanzapine, also positive effects on stuttering have been reported. In a study of 23 patients, olanzapine proved to be better than placebo at reducing symptoms of stuttering (10). Alaghband-Rad et al. (11) describe two children with an autistic disorder in which (worsening of) stuttering occurred during memantine (a glutamate inhibitor) treatment. Mancano (12) describes a case of pregabalin (an antiepileptic interfering in the GABA pathway as well)-induced stuttering in a 31-year-old woman. Atay et al. (13) describe a patient who started to stutter after chronic use of a low dose of risperidone (an antipsychotic interfering mainly with the serotonin and dopamine pathways). Yadav (14) also describes a patient who began to stutter after taking risperidone. In this case, there was a clear dose-dependent effect of risperidone on stuttering. That risperidone causes stuttering is paradoxical, as risperidone is also used in the treatment of stuttering (15, 16). Furthermore, bupropion (7), lithium (17), and sertraline (18–20) have also been linked to the (re)occurrence of stuttering.

Moreover, the antimuscarinic properties of tricyclic antidepressants (TCAs) have been related to provoking or aggravating stuttering (4). Hays (21) reported an increase in speech disorders in depressed patients who stuttered and were treated with TCAs. Based on this observation, he set up a study into the effect of bethanechol (a cholinergic substance) in two bipolar patients. Their speech became more fluent with the use of bethanechol. Kampman et al. (22) also reported positive effects of bethanechol on stuttering.

Speech and language disorders are common in children with ADHD. Studies show that 15–30% of children with ADHD have speech and language disorders (23). Briley and Ellis (24) suggest a possible relationship between stuttering and ADHD. Children who stutter have a higher odds of ADHD (odds ratio = 3.1, 95% CI 2.6–3.7). This could be due to the fact that certain characteristics are more present in children who stutter, such as inattention and hyperactivity/impulsivity (24). Five drugs in the top 20 drug–stuttering associations with the highest disproportionality in VigiBase are drugs prescribed for the treatment of ADHD (Table 1). The paradoxical usage of the very same drug methylphenidate (acting *via* the dopamine pathway) in experimental treatment of stuttering (25) is dealt with below.

In the general population of developmental stutterers, men appear to stutter more frequently than women. In VigiBase, however, women are overrepresented (57% of the patients were female, 39% male, 4% unknown). This may indicate that most cases of drug-induced stuttering indeed are of the acquired type—in contrast to developmental stuttering, where a strong male predominance is found (1).

### Mechanisms of Drug-Induced Stuttering

Several mechanisms have been associated with the development of drug-induced stuttering. Increased dopamine levels, reduction of GABA, anticholinergic properties of drugs, or changes in serotonin levels are factors that may contribute to the development of stuttering.

Because some studies found that stuttering symptoms can be reduced by dopamine antagonists [e.g., haloperidol (26) and risperidone (16)] while increased by dopamine stimulant, the possible role of dopaminergic mechanisms is suggested.

A hyperdopaminergic state was observed in stuttering patients in the medial prefrontal cortex, ventral limbic cortical regions, subcortical regions, and temporal cortical regions (including the auditory cortex) (27). As the medial prefrontal cortex is functionally connected to the supplementary motor area, which plays an important role in speech motor control circuits, some researchers have proposed a dopamine excess hypothesis of stuttering (10, 27–29). According to this hypothesis, stuttering is related to abnormal elevations of cerebral dopamine activity. However, further research on the anatomical and functional changes in the dopaminergic pathways in stuttering patients is needed (30).

However, this notion is not without controversy, as these effects by dopamine antagonistic antipsychotics might also be mediated by anticholinergic properties of antipsychotics (4). It was observed that antipsychotics can provoke and suppress stuttering. Therefore, it was hypothesized that a change in dopamine/acetylcholine balance can also have an effect on stuttering in susceptible people (4).

Relevant aspect in this paradoxical effect of the dopamine pathway in stuttering is the inverted U-shaped effect of dopamine in cognitive control, described by Cools and D'Esposito (31) in 2011. Optimum dopamine levels for cognitive functions vary within people as well as within tasks, and manipulating dopamine may have therefore paradoxical effects in different processes. This then may underlie the challenges seen in experimental therapeutical approaches (the balance between effects and side effects is not easily met) and may explain the abovementioned paradoxes in provoking or suppressing stuttering with the very same drugs, for instance, methylphenidate.

Quite a lot of reports involved selective serotonin reuptake inhibitors (SSRIs) as suspect drugs. This suggests that serotonergic mechanisms might play a role. Also, akathisia is commonly present in cases of drug-induced stuttering. The SSRIs are more prone to produce akathisia than most other antidepressants (4). Serotonin might bring about akathisia by the inhibition of dopamine pathways in the nigrostriatal. A similar mechanism may be operating in serotonin-induced stuttering (4).

Stuttering is a rare side effect of theophylline therapy, what may also give insight into the mechanisms involved in stuttering. Theophylline-induced stuttering is hypothesized to involve the disruption of the optimal balance between excitatory and inhibitory neurotransmission throughout the brain by inhibiting GABA receptors. This may lead to hyperexcitation of the motor cortex that may mimic the motor cortex hyperexcitability that exists in developmental stuttering (30).

### Treatment of Drug-Induced Stuttering

Market et al. (32) described the favorable response on mainstream stuttering-specific procedures in 81 people with acquired stuttering they could trace. Within that total number, drug-induced stuttering was found in 6.2% and the therapeutic response was not reported specifically in that group. Generally, one would try to remove or adjust the putative causal drug. Therapy remains a process of trial and error.

### Strength and Limitations of the Study

This is a study of drug-induced stuttering, searching for the effect of the totality of drugs, as reported in the worldwide VigiBase. Quite recently, a similar study was published that looked at disproportionality in VigiBase using the MedDRA LLTs “stutter” and “stuttering.” They found that clozapine, pregabalin, olanzapine, methylphenidate, adalimumab, and risperidone were the most frequently implicated drugs, whereas they found the highest ROR values with methylphenidate, topiramate, olanzapine, golilumab, clozapine, and pregabalin (33). These results are very similar but not totally identical, possibly caused by a slightly different search strategy.

Some drugs have a high value for the ROR with, however, a broad 95% CI. This is the case when the number of reports for the association is low. Therefore, it is important to keep in mind that the ROR is a measure of disproportionality, not of causality.

Acquired stuttering can be hard to distinguish from related speech and language disorders, such as dysarthria (8). This may result in misclassification when coding these speech disorders for inclusion in the database.

No distinction between the development of stuttering and aggravation of stuttering could be made based on the coded information on the reports, since there are no separate MedDRA terms to code this. Furthermore, in-depth information as reported in the narrative was not available to the authors.

Another limitation is the lack of information on many of the reports in VigiBase. Missing data means that it may be difficult to make a clinical judgment regarding case relevance (34).

Obviously, these VigiBase data cannot be used to give an indication as to the frequency of side effects (here stuttering) in relation with the drugs used. Usually, such quantitative relationships are being found in prospective studies or controlled clinical studies.

## Conclusion

Based on a search *via* the international pharmacovigilance database VigiBase, a wide variety of drugs have been linked to the occurrence or recurrence of stuttering. Case reports were discussed, taking into account the mechanisms of the pharmacologic agents being used. Different neurotransmitter systems seem to play a role in the development of stuttering. The discussion of these different systems is complicated, however, by the fact that many of the reported drugs are promiscuous in their interaction with different neurotransmitter receptors and/or transporters. Examples pertaining to some of the drugs mentioned in the tables are quoted here: duloxetine (35), pregabalin (36), methylphenidate and amphetamine (37), and bupropion (38). Paradoxically, agents known to reduce stuttering in some people may induce it in others. These data are discussed, taking into account both qualitative and quantitative aspects of neurotransmitters and interfering substances. The inverted U-shaped effect of dopamine in cognitive control, described by Cools and D'Esposito (31) in 2011, may be crucial in this paradoxical effect. These adverse drug reactions (mostly seen in psychopharmacotherapy) may help to study the pharmacological pathways of normal speech as well as of stuttering. Increased knowledge in these pathways seems to be required before devising productive pharmacological interference with stuttering.

## Data Availability Statement

The VigiBase datasets generated and analysed during the current study are not publicly available due to agreements between contributors of data to the database used (VigiBase) and the custodian of this database. National centres (mainly national drug regulatory authorities) constituting the WHO Programme for International Drug Monitoring (PIDM) contribute data to VigiBase, and UMC is the custodian in its capacity as a WHO collaborating centre for international drug monitoring.

## Author Contributions

BB, CE, and FH contributed to the conception and the design of the review. CE and FH organized the database. BB, PvH, and TY wrote sections of the manuscript. All authors contributed to the manuscript revision, read, and approved the submitted version.

## Author Disclaimer

The information presented here does not represent the opinion of the UMC or the World Health Organization.

## Conflict of Interest

The authors declare that the research was conducted in the absence of any commercial or financial relationships that could be construed as a potential conflict of interest.

## Publisher's Note

All claims expressed in this article are solely those of the authors and do not necessarily represent those of their affiliated organizations, or those of the publisher, the editors and the reviewers. Any product that may be evaluated in this article, or claim that may be made by its manufacturer, is not guaranteed or endorsed by the publisher.
